# Yoda1 Enhanced Low-Magnitude High-Frequency Vibration on Osteocytes in Regulation of MDA-MB-231 Breast Cancer Cell Migration

**DOI:** 10.3390/cancers14143395

**Published:** 2022-07-13

**Authors:** Chun-Yu Lin, Xin Song, Yaji Ke, Arjun Raha, Yuning Wu, Murtaza Wasi, Liyun Wang, Fei Geng, Lidan You

**Affiliations:** 1 Institute of Biomedical Engineering, University of Toronto, Toronto, ON M5S 3G9, Canada; delphine.lin@mail.utoronto.ca (C.-Y.L.); yaji.ke@mail.utoronto.ca (Y.K.); 2 Mechanical and Industrial Engineering, University of Toronto, Toronto, ON M5S 3G8, Canada; suzie.song@mail.utoronto.ca; 3 W Booth School of Engineering Practice and Technology, McMaster University, Hamilton, ON L8S 4L7, Canada; rahaa@mcmaster.ca (A.R.); wuy198@mcmaster.ca (Y.W.); gengf@mcmaster.ca (F.G.); 4 Department of Mechanical Engineering, University of Delaware, Newark, DE 19716, USA; murtaza@udel.edu (M.W.); lywang@udel.edu (L.W.)

**Keywords:** low-magnitude high-frequency vibration, vibration, Piezo1, Yoda1, osteocyte, breast cancer, bone metastasis, osteoclastogenesis, mechanical stimulation, YAP

## Abstract

**Simple Summary:**

Bone metastasis is a severe complication that contributes significantly to the morbidity and mortality of breast cancer patients. Although mechanical loading was shown to mediate bone remodeling and attenuate metastatic tumor growth, commonly prescribed exercise can be physically challenging for cancer patients. Low-magnitude high-frequency (LMHF) vibration as an exercise alternative enhances bone mineral density while being safe and easy-to-perform. However, vibration alone appears to be insufficient in reducing bone loss caused by breast cancer. Yoda1, an activator of the mechanosensitive Piezo1 channel, provides a potential solution to intensify the effects of LMHF vibration. This study aims to investigate the effects of combined treatment (Yoda1 and LMHF vibration) in regulating osteoclastogenesis and breast cancer cell migration. We confirmed that combining LMHF vibration and Yoda1 reduces the formation of osteoclasts and further inhibits the migration of MDA-MB-231 breast cancer cells. Our data supported the novel strategy to regulate cancer cell migration for high-risk patients.

**Abstract:**

Low-magnitude (≤1 g) high-frequency (≥30 Hz) (LMHF) vibration has been shown to enhance bone mineral density. However, its regulation in breast cancer bone metastasis remains controversial for breast cancer patients and elder populations. Yoda1, an activator of the mechanosensitive Piezo1 channel, could potentially intensify the effect of LMHF vibration by enhancing the mechanoresponse of osteocytes, the major mechanosensory bone cells with high expression of Piezo1. In this study, we treated osteocytes with mono- (Yoda1 only or vibration only) or combined treatment (Yoda1 and LMHF vibration) and examined the further regulation of osteoclasts and breast cancer cells through the conditioned medium. Moreover, we studied the effects of combined treatment on breast cancer cells in regulation of osteocytes. Combined treatment on osteocytes showed beneficial effects, including increasing the nuclear translocation of Yes-associated protein (YAP) in osteocytes (488.0%, *p* < 0.0001), suppressing osteoclastogenesis (34.3%, *p* = 0.004), and further reducing migration of MDA-MB-231 (15.1%, *p* = 0.02) but not Py8119 breast cancer cells (4.2%, *p* = 0.66). Finally, MDA-MB-231 breast cancer cells subjected to the combined treatment decreased the percentage of apoptotic osteocytes (34.5%, *p* = 0.04) but did not affect the intracellular calcium influx. This study showed the potential of stimulating Piezo1 in enhancing the mechanoresponse of osteocytes to LMHF vibration and further suppressing breast cancer migration via osteoclasts.

## 1. Introduction

Bone metastasis, the spread of cancers to the bone, is presented in 80% of advanced breast cancer patients [1]. Severe symptoms, including osteolysis and pathological fractures, result in a significant reduction in the longevity and quality-of-life of patients with limited treatment options. For osteolytic bone metastasis, the extravasation of cancers to the skeleton disrupts the bone homeostasis between osteoblasts (bone-forming cells) and osteoclasts (bone-resorbing cells), eventually leading to an increase in bone resorption and a “vicious cycle” that promote aggressive cancer growth [2]. In addition, mechanical stimulation generated through exercises also highly regulates bone homeostasis. Studies thus have begun to explore the potential of biomechanical approaches as a treatment to counteract tumor-mediated osteolytic destruction [3,4,5].

The ability of carefully controlled high-magnitude mechanical loads to suppress breast cancer growth and maintain bone integrity has been shown using various models in vivo. In an experiment on MDA-MB-231 metastatic breast cancer-injected immunocompromised mice, dynamic tibial compression (4.1 N, 600 μstrains, 4 Hz) dramatically reduced tumor formation and tumor-driven bone degradation [3]. Beneficial effects, including increased bone volume ratio, reduced trabecular degradation, and enhanced bone mineral density (BMD), were found in mice injected with EO771 and 4T1.2 epithelial-like breast cancer cells after tibial loading (1 N, 260 μstrains, 2 Hz) [4]. Moreover, moderate treadmill running and tibial loading (4.5 N, 630 μstrains, 4 Hz) significantly inhibited tumor proliferation and tumor-mediated osteolysis in Py8119 triple-negative breast cancer cell-inoculated mice [5]. However, the high-magnitude stimulation generated through commonly prescribed exercise regimens could be physically challenging for frail cancer patients and can increase their risk of accidental injury.

Whole body vibration (WBV) at low-magnitude (≤1 g) high-frequency (≥30 Hz) (LMHF) has been proposed as an exercise alternative for cancer patients in contrast to interventions involving high magnitude mechanical forces [6]. The procedure is non-invasive, safe, and easy-to-perform. Studies have shown the anabolic benefits of LMHF vibration (LMHFV) on the musculoskeletal system, including increased bone density [7], reduced marrow fat [8], and improved muscle and glucose metabolism [9]. At the cellular level, vibratory stimuli have been shown to enhance the osteogenic response by promoting the growth of osteoblasts and inhibiting osteoclasts [10,11]. 

Nonetheless, some clinical studies have demonstrated no positive effect or significant difference in the bone quality of breast cancer patients and postmenopausal women after WBV treatment [12,13], making its implementation in these populations controversial. A meta-analysis suggested that there is no improvement in the BMD of the femoral neck for postmenopausal women after 3–13 months of WBV [12]. A randomized clinical trial showed that WBV is safe but insufficient for preventing bone loss in breast cancer patients receiving aromatase inhibitors [13]. Thus, strategies for intensifying the effect of LMHF vibration on bone cells, especially in frail and older patients, are needed to bridge the gap.

Piezo1 protein is a mechanosensitive ion channel that allows intracellular calcium influx in response to various mechanical signals such as static pressure, shear stress, and membrane stretch and further initiates downstream biological responses [14]. It is critical in various physiological processes [15], including the development of vasculatures, regulation of red blood cell volume, and sensation of light touch. Piezo1 is also highly expressed in the bone, regulating the differentiation of mesenchymal stem cells and mediating calcium ion influx in osteocytes and osteoblasts [16,17]. Mice with conditional deletion of Piezo1 in osteogenic lineage cells exhibited osteopenia and blunted response to treadmill running and tibial loading [16,18]. Mice and newborn mice with Piezo1 deficiency in osteoblastic cells showed loss of bone mass and increased bone resorption [16,18,19]. The evidence suggests the importance of Piezo1 in bone development. Yoda1, as a Piezo1 agonist, selectively activates Piezo1 in the absence of externally applied pressure [20]. It was shown that the injection of Yoda1 promotes bone formation in young adult mice [18]. Thus, Yoda1 provides the potential to elevate the mechanoresponse of the bone cells.

Osteocytes, the most abundant cells in the bone, play a crucial role in maintaining balanced bone homeostasis through the crosstalk with osteoblasts and osteoclasts. Osteocytes are also the principal mechanosensors in the bone, sensing the surrounding mechanical cues and initiating mechanotransduction to further mediate the communication among effector cells. Mechanically stimulated osteocytes have been demonstrated to have anti-metastatic and anti-resorptive potentials. It was shown that oscillatory fluid flow (OFF) (1 Pa, 1 Hz)-stimulated osteocytes inhibited invasion and induced apoptosis of breast cancer cells through osteoclasts or endothelial cells [21]. Extravasation of MDA-MB-231 across endothelial cells toward osteocytes was shown to significantly reduce when applying OFF (1 Pa, 1 Hz) to osteocytes [22]. Moreover, osteocytes responded to vibration with increased gap junctional intracellular communication [23] and altered transcriptional expression levels that favor anti-resorptive effects [24]. Due to the anti-metastatic and anti-osteolytic potentials, mechanically stimulated osteocytes have become the target of the current study. Their high mechanical sensitivity and abundance could also plausibly benefit patients with low Yoda1 concentration and the low resulting side effects.

Although osteocytes are the target of the current study, the direct effects of vibration and Yoda1 on other cell types are unknown and may play an essential role in vibration regulation of breast cancer bone metastasis. Both treatments systemically affect other cell types in the metastatic microenvironment in the in vivo models. Numerous studies have investigated the effects of LMHF vibration and the roles of Piezo1 on breast cancer cells separately. Application of LMHF vibration on MDA-MB-231 cells does not affect their migration [25], cell viability, and apoptosis but suppresses their invasion and upregulates FAS, a membrane death receptor [26]. Piezo1 channels are overexpressed in MCF-7 breast cancer cells and are also shown to contribute to the migration, proliferation, and invasion of several cancer cell lines [14]. However, the effects of Yoda1 on MDA-MB-231 breast cancer cells and RAW264.7 osteoclast precursors remain unknown. Thus, we investigated the dosage effects of Yoda1 on cells and performed combined treatment (Yoda1 and LMHF vibration) on breast cancer cells in the regulation of osteocytes.

In this study, we hypothesize that the stimulation of Piezo1 with Yoda1 enhances the mechanoresponse of osteocytes to LMHF vibration, inhibiting the formation of osteoclasts and further reducing the migration of breast cancer cells. Since the dosage effects of Yoda1 remain unclear in the principal cell types of the current study, we first examined the direct effects of Yoda1 on MLO-Y4 osteocyte-like cells, MDA-MB-231 breast cancer cells, and RAW264.7 osteoclast precursors. We also measured the migration of MDA-MB-231 under LMHF vibration. To test our hypothesis, we observed nuclear translocation of YAP (Yes-associated protein), an important transcriptional regulator related to mechanical activation, in osteocytes under combined treatment (Yoda1 and LMHF vibration). We investigated the effects of osteocyte’s response to combined treatment on breast cancer migration through indirect signaling of osteoclasts. Finally, we tested how soluble factors released by breast cancer cells under combined treatment may mediate the quantity and apoptosis of osteocytes. Overall, results from this study show that combined treatment can regulate MDA-MB-231 cancer cell migration, suggesting a safe and effective strategy for breast cancer bone metastasis patients with a low tolerance to physical and pharmaceutical interventions to maintain bone integrity and homeostasis.

## 2. Materials and Methods

### 2.1. Cell Cultures

MLO-Y4, MDA-MB-231, Py8119, and RAW264.7 were used in this study. MLO-Y4 (a gift from Dr. Bonewald, Indiana University) is a murine osteocyte-like cell line. They were seeded on tissue culture dishes coated with type I rat tail collagen (354249, VWR, Radnor, PA, USA) and maintained in MLO-Y4 medium (94% α-MEM (12571, Gibco), 2.5% calf serum (CS, all Thermo Fisher Scientific, Waltham, MA, USA), 2.5% fetal bovine serum (FBS, all Gibco, Thermo Fisher Scientific, Waltham, MA, USA), 1% penicillin streptomycin (P/S, Gibco)). Cells that reached 80% confluency were detached and passaged using Trypsin-EDTA (25200072, Gibco). Prior to mono- (Yoda1 only or vibration only) or combined treatment (Yoda1 and LMHF vibration), MLO-Y4 cells were seeded in collagen-coated 35 mm × 10 mm tissue culture dishes (353001, Corning Inc., Corning, NY, USA) at a density of 1.5 × 10^5^ cells/well for two days to ensure 80% confluency was reached.

MDA-MB-231 (HTB-26, all ATCC, Manassas, VA, USA) metastatic human breast cancer cells were maintained in MDA-MB-231 medium (F-12K (Gibco) with 10% FBS and 1% P/S) in tissue culture dishes. To collect the secreting factors, cells were seeded at a density of 2 × 10^5^ cells/well in 35 mm × 10 mm tissue culture dishes for two days to reach 90% confluency. Then, the medium that contained soluble cytokines secreted by the cells was extracted as conditioned medium (CM) for further study (non-stimulated MDA-MB-231 CM). Py8119 (CRL-3278, ATCC) murine breast cancer cells were maintained in Py8119 medium (F-12K with 5% FBS and 1% P/S) in tissue culture dishes.

RAW264.7 (TIB-71, ATCC) cell line was differentiated into osteoclasts. They were maintained in RAW medium (87% DMEM (D5671, all Sigma-Aldrich, St. Louis, MO, USA), 10% FBS, 1% P/S, 2% L-glutamine (25030081, Gibco)) in tissue culture dishes. Cells were passaged using cell scrapers and replated to fresh dishes. All cells were maintained at 37 °C and 5% CO_2_.

### 2.2. Low-Magnitude High-Frequency Vibration

**Figure 1:**
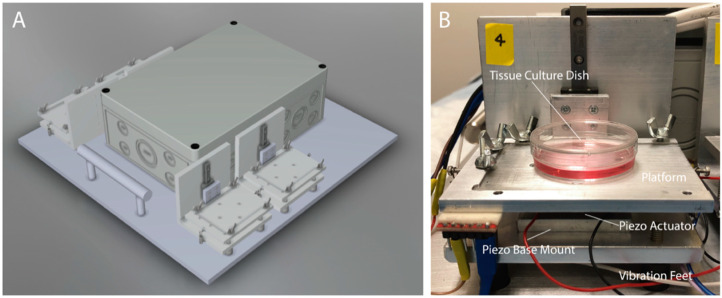
The custom LMHF vibration system. (**A**) Overview of the vibration system. (**B**) Description of the components in the vibration platform. Size = 85 mm × 76 mm × 94 mm.

The custom LMHF vibration system (Figure 1) consists of four individually adjustable vibration platforms that allow vertical vibrations with amplitudes and frequencies of 0.1–0.3 g and 30–90 Hz, respectively. The high-throughput, humidity-resistant, and user-friendly vibration system is used to generate LMHF vibrations for incubated cells. 

To provide mono- or combined treatment on cells, MLO-Y4 or MDA-MB-231 cells were washed with phosphate buffered saline (PBS) twice and were treated with 0.1% *v*/*v* DMSO or 10 μM Yoda1 (SML1558-5MG, Sigma-Aldrich) for the total duration of 2 or 4 h. During the second hour of incubation, cells were subjected to 0.3 g, 60 Hz vibration for 1 h. Static groups were placed in the incubator. Immediately after the treatment, cells were lysed for Western blot study and fixed for immunofluorescence. For CM collection (stimulated MDA-MB-231 CM or MLO-Y4 CM), all groups were incubated with 1 mL of fresh growth medium, which was collected 1 h later to allow sufficient secretion of growth factors. 

### 2.3. Live-Dead Staining

MLO-Y4, MDA-MB-231, or RAW264.7 cells were seeded in 48-well plates (day 0) and treated with DMSO or Yoda1 (day 1). Cell seeding density differed between different durations of studies to avoid over confluence at the endpoint. After 2, 24, or 48 h incubation, cells were stained and incubated with 20 μL NucBlue^®^ Live (Ex: 360 nm, Em: 460 nm, all Invitrogen, Waltham, MA, USA) and 20 μL NucGreen^®^ Dead (Ex: 504 nm, Em: 523 nm, Invitrogen) stain for 30 min, following by capturing three random images per well using the fluorescence microscope (Nikon, Minato City, Tokyo, Japan). The images were analyzed using ImageJ to determine the number of cells. NucBlue^®^ Live stained all cells, while NucGreen^®^ Dead stained only dead cells. Positive control (PC) was prepared by treating cells with 70% ethyl alcohol for 15 min prior to staining. 

### 2.4. BrdU Cell Proliferation Assay

Cell proliferation was measured using the BrdU Cell Proliferation ELISA kit (ab126556, Abcam, Cambridge, UK) according to the protocol provided by the manufacturer. The BrdU incorporation was measured using the spectrophotometric microtiter plate reader at a dual wavelength of 450/550 nm.

### 2.5. Apoptosis Assay

MDA-MB-231 cells were plated in 48-well plates (day 0) and treated with DMSO or Yoda1 (day 1) for 24 or 48 h. Apoptosis of these cells was quantified by incubating with 5% APOPercentage dye (A1000, Biocolor, Carrickfergus, UK) for 30 min, followed by PBS wash. Six random images per well were captured under the microscope for quantification to identify the apoptotic cells that were stained in pink.

To examine the apoptotic effect of breast-cancer-derived factors on osteocytes, MLO-Y4 cells were seeded in collagen-coated 48-well plates at a density of 5 × 10^3^ cells/well. After 24 h, the medium was replaced with 50% MLO-Y4 medium and 50% non-stimulated or stimulated MDA-MB-231 CM (or 50% MLO-Y4 medium and 50% fresh MDA-MB-231 medium) for three consecutive days. In addition, the same procedures for apoptosis quantification were performed.

### 2.6. Scratch Assay

A scratch assay was performed to quantify the rate of cell migration. A sample of the MDA-MB-231 cells was seeded in a 48-well plate, and the test was conducted when it formed a monolayer. The scratches were created using a 200 μL pipette tip. Reference points on the outer bottom of the plates were established using an ultrafine marker to enable a consistent field of images during image acquisition. For the dosage study, either DMSO or Yoda1 was added immediately after scratch creation. For investigating the effects of LMHF vibration, cells were subjected to LMHF vibration of 0.3 g, 60 Hz for 1 h immediately after scratch creation. Then, the images were acquired at 0, 4, 12, or 24 h using a phase-contrast microscope. Three regions of view on a single scratch were captured. The scratch area was determined using Image J and Wound Healing Size Tool plugin [27]. *Wound closure* % was defined according to the following equation [28]. *A_t_*_= 0_ is the initial wound area and *A_t_*_= Δ*t*_ is the wound area after t hours:

$$Wound\;Closure\;\%=\left(\frac{A_{t=0}-A_{t=\Delta t}}{A_{t=0}}\right)\times 100\%$$

### 2.7. Osteoclastogenesis

RAW264.7 osteoclast precursors were seeded at a density of 1 × 10^4^ cells/well in 24-well plates in RAW medium. After 48 h, cells were subjected to dose-dependent Yoda1 every subsequent day until day 5. An additional 20 ng/mL RANKL (462-TR-010, R&D Systems Inc., Minneapolis, MN, USA) was added to induce osteoclastogenesis along with the treatment. On day 6, the medium was replaced with RAW medium. On day 7, cells were fixed and stained with tartrate-resistant acid phosphatase (TRAP). Five fields of view were captured randomly under the microscope for osteoclast quantification, and the osteoclasts were identified as TRAP-positive multinucleated cells.

To identify the effect of MLO-Y4 CM on osteoclastogenesis, the similar protocol was repeated for 5-day differentiation. The medium was replaced daily with the mixture of 50% MLO-Y4 CM and 50% growth medium supplemented with 20 ng/mL RANKL for three consecutive days after seeding. On day 4, the medium was replaced with fresh RAW264.7 medium to ensure MLO-Y4-derived factor was not collected in CM. The CM of RAW264.7 (RAW264.7 CM) was collected at the endpoint for transwell study, and osteoclastogenesis was measured as described using TRAP.

### 2.8. Immunoblotting

Cell pellets were washed by 1X PBS (1610780, Bio-Rad, Hercules, CA, USA) and resuspended in 50 μL cell lysis buffer (with 1% Triton X-100, 50 mM Tris-HCl, pH 8.0, 100 mM NaCl, 1 mM EDTA) that was supplemented with protease inhibitor (05892970001, Roche, Basel, Switzerland). The protein concentration of the cell lysates was determined using Pierce™ BCA Protein Assay Kit (23227, Thermo Fisher Scientific) and Tecan microplate reader (Infinite M200 Pro, Tecan, Männedorf, Switzerland). 

The expression level of YAP in the cell lysates was measured by capillary electrophoresis-based immunoassay (AY2093, Abby, all ProteinSimple, San Jose, CA, USA). Cell lysates were diluted to a concentration of 0.5 μg/μL using 0.1 X sample diluent buffer and 5 × fluorescent master mix that were provided in the Training Kit (PS-T006, ProteinSimple). Then, the derived sample mixtures were heated at 95 °C for 5 min prior to the immunoassay. The primary antibodies for the detection of β-actin and YAP were as follows: β-actin (8H10D10, all Cell Signaling, Danvers, MA, USA) mouse mAb (3700S, Cell Signaling) and YAP (D8H1X, Cell Signaling) XP^®^ rabbit mAb (14074S, Cell Signaling). The secondary antibodies for β-actin and YAP were also included in the Training Kit (PS-T006, ProteinSimple), and they were anti-mouse secondary antibody (PART NO: 042-205) and anti-rabbit secondary antibody (PART NO: 042-206), respectively. Protein sample mixtures, primary antibodies, and other required reagents were dispensed into an assay plate (supplied in the Training Kit) according to the manufacturer’s protocol. The assay module that was used in this experiment was the 12–230 kDa Abby Separation Module and 8 × 25 capillary cartridges (SM-W004, ProteinSimple). Default assay parameters were applied to the run, and the results were evaluated with the Compass for Simple Western software v6.1 (Biotechne, Minneapolis, MN, USA). 

The peak area values were used to represent the intensity of target proteins. YAP intensity was first normalized against the corresponding β-actin intensity. Then, the relative YAP expression of mono- or combined treatment groups were further normalized to the DMSO control.

### 2.9. Immunofluorescent Staining

All the cells were fixed with 4% paraformaldehyde and permeabilized with lysis buffer (0.1% Triton X-100 in PBS). Then, the cells were incubated with blocking buffer (3% BSA in PBS) before the subsequent overnight incubation with mouse anti-YAP (sc-101199, Santa Cruz Biotechnology, Dallas, TX, USA) at a dilution of 1:2000. Samples were washed and incubated with secondary antibodies, fluorescein isothiocyanate (FITC) conjugated goat anti-Mouse antibody (F0257, Sigma-Aldrich), at room temperature for 1 h. Cell nuclei of each sample were then stained with DAPI (D1306, Thermo Fisher Scientific) solution at room temperature for 10 min prior to the subsequent fluorescent microscopy (IX51S1F-3, Olympus, Shinjuku City, Tokyo, Japan).

### 2.10. Transwell Migration Assay

Cells were first stained with cell tracker green (C2925, Thermo Fisher Scientific) in no serum medium (99% F-12K, 1% P/S) for 40 min. Then, the dye was replaced with fresh growth medium for 30 min to allow cell recovery. In a 12-well plate, 1200 µL of RAW264.7 CM was added to each well, and ThinCert^®^ cell culture inserts (665638, Greiner Bio-One, Kremsmünster, Austria) were placed on the top. Breast cancer cells were seeded at 4 × 10^5^ cells/well density and allowed to migrate for 6 h. At the endpoint, non-migratory cells located on the upper compartment of ThinCert^®^ were removed using a cotton swab, and the remaining cells were fixed with 10% neutral buffered formalin (HT501128, Sigma-Aldrich). Five images per insert were taken under the fluorescence microscope, and the mean fluorescent intensity was further quantified using Image J. Data points that exceeded the mean ± 1.5 times of standard deviation were defined as outliers. Outliers were deleted within the groups and after combining all groups. All groups were normalized to DMSO control.

### 2.11. Intracellular Calcium Imaging of MLO-Y4 Cells under Oscillatory Fluid Flow

MLO-Y4 cells were seeded at 6 × 10^4^ cells/mL density in Type-I collagen-coated μ-Slide (80606, ibidi, Gräfelfing, Germany) and subjected to 50% MLO-Y4 medium and 50% stimulated/non-stimulated MDA-MB-231 (or 50% MLO-Y4 medium and 50% fresh MDA-MB-231 medium) for three consecutive days. On the day of measurement, the cells were stained with Fura-2 AM (Ex: 340/380 nm, Em: 510 nm, Thermo Fisher Scientific) for 40 min at room temperature and were rinsed with working medium (98% α-MEM without phenol red, 1% FBS, and 1% P/S). The μ-Slide was then connected to the syringe pump for the parallel plate flow chamber to generate OFF (2 Pa, 1 Hz). Cells were imaged using PTI EasyRaitoPro (Horiba, Kyoto, Kyoto, Japan). For the first minutes, a static baseline reading of cell response was taken, at *t* = 1 min, and flow was applied to the osteocytes for 3 min. After the experiment, the ratiometric calcium data (340/380 nm ratio) was analyzed using a previously developed MATLAB code [29] to determine the response rate and mean magnitude.

### 2.12. Statistics

All statistical analyses were performed using GraphPad Prism 9 (GraphPad Software, San Diego, CA, USA). All experiments were repeated independently at least twice with at least three samples for each group in one experiment. Data points from the representative trail of experiments or all experiments are presented as mean + standard deviation. For the comparison between two groups, two-tailed Student’s *t*-test was performed to define statistical significance. For the comparison between more than two groups, ordinary one-way analysis of variance (ANOVA) or two-way ANOVA followed by Tukey’s multiple comparisons were conducted to define statistical significance. Significance was taken at α = 0.05.

## 3. Results

### 3.1. Live-Dead Measurements of MLO-Y4 and MDA-MB-231 under Yoda1 Treatment

**Figure 2:**
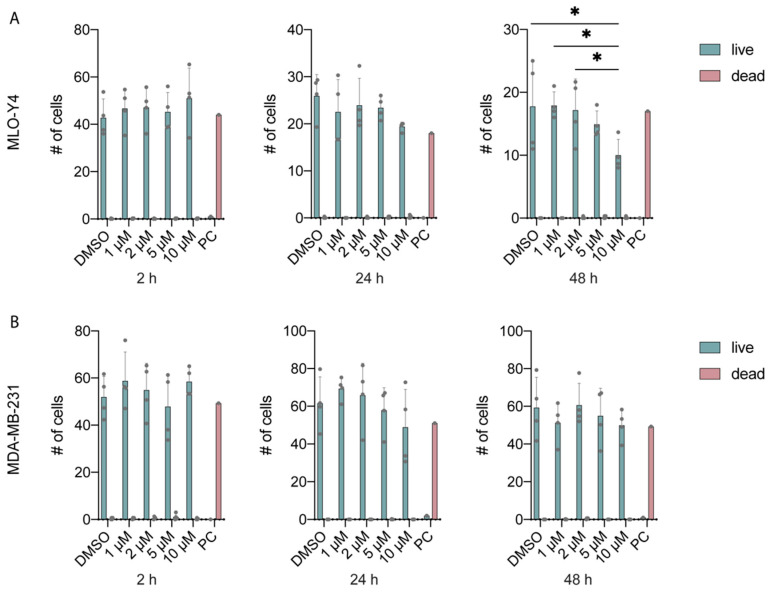
Live-dead measurements of MLO-Y4 and MDA-MB-231 under dose-dependent Yoda1 treatments for 2, 24, and 48 h. (**A**) Live-dead measurements of MLO-Y4, (**B**) live-dead measurements of MDA-MB-231 after subjected to DMSO or 1, 2, 5, 10 μM Yoda1 for 2, 24, and 48 h (*n* = 4 for DMSO and Yoda1 treatments, *n* = 1 for PC). PC: positive control. * *p* < 0.05.

To investigate the survival of osteocytes and breast cancer cells under Yoda1, we compared the live-dead measurements of MLO-Y4 and MDA-MB-231 under dose-dependent Yoda1 for 2, 24, and 48 h (Figure 2). The change in number of live MLO-Y4 remained non-significant under Yoda1 treatments for 2 and 24 h. Although 10 μM Yoda1 significantly reduced the number of live MLO-Y4 by 43.6% compared to DMSO at 48 h (Figure 2A), the dead cells remained low. The result might be due to the decrease in proliferation under a high concentration of Yoda1 treatment, or the cells were removed during PBS wash prior to imaging. No significant difference was detected for MDA-MB-231 in numbers of live and dead cells for all time points (Figure 2B).

### 3.2. Direct Effects of Yoda1 or LMHF Vibration on MDA-MB-231 or RAW264.7

**Figure 3:**
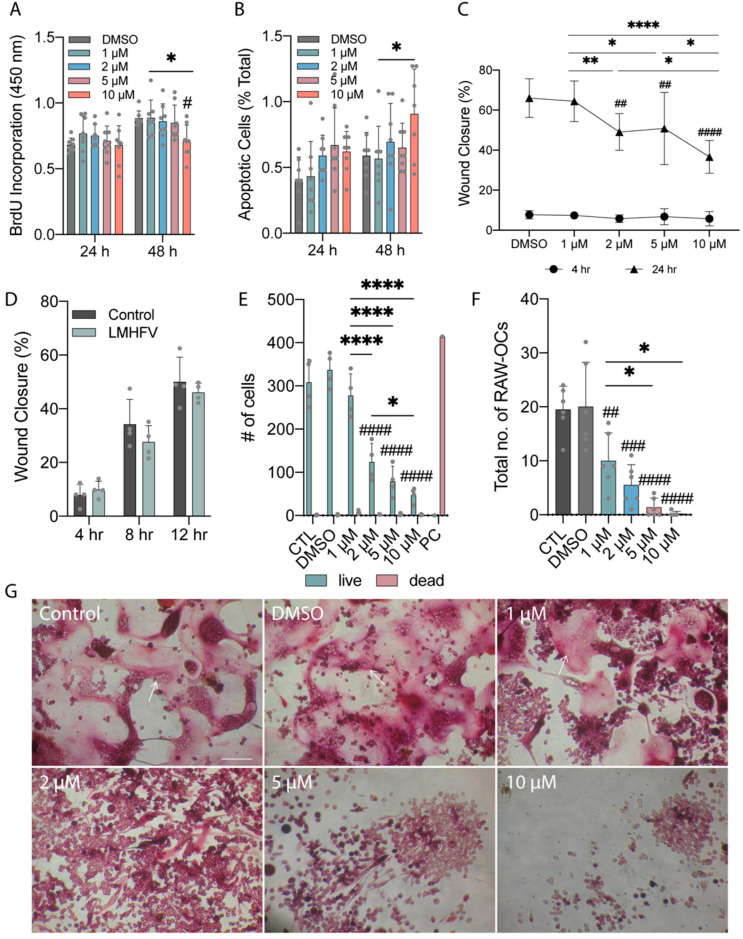
Yoda1 treatments suppressed proliferation and migration of breast cancer cells and reduced osteoclastogenesis. (**A**) Cell proliferation of MDA-MB-231 and (**B**) percentage of apoptotic MDA-MB-231 after subjected to DMSO or 1, 2, 5, 10 μM Yoda1 for 24 and 48 h (*n* = 8). (**C**) Migration of MDA-MB-231 after subjected to DMSO or 1, 2, 5, 10 μM Yoda1 for 4 and 24 h (*n* = 8). (**D**) Migration of MDA-MB-231 at 4, 8, and 12 h after subjected to vibration of 0.3 g, 60 Hz for 1 h (*n* = 4). (**E**) Live-dead measurements of RAW264.7 under medium (Control), DMSO, or dose-dependent Yoda1 for 48 h (*n* = 4 for DMSO and Yoda1 treatments, *n* = 1 for PC). PC: positive control. (**F**) Total number of TRAP-positive RAW-OCs with more than 2 nuclei after 7-day incubation in medium (Control), DMSO, or dose-dependent Yoda1 supplemented with 20 ng/mL RANKL (*n* = 6). (**G**) Representative images of multinucleated RAW-OCs for the groups described in (**F**). Arrows indicate the representative TRAP-positive RAW-OCs. Scale bar = 100 μm. **** *p* < 0.0001, ** *p* < 0.01, * *p* < 0.05; ^####^ *p* < 0.0001, ^###^ *p* < 0.001, ^##^ *p* < 0.01, ^#^ *p* < 0.05 in comparison to DMSO control.

We further evaluated the roles of Yoda1 and LMHF vibration on breast cancer cells. We first incubated MDA-MB-231 with Yoda1 and measured the resulting proliferation, apoptosis, and migration. Then, we measured the migration of MDA-MB-231 after 1 h treatment of LMHF vibration at 0.3 g, 60 Hz. When treated with a high concentration of Yoda1 (10 μM) for 48 h, breast cancer proliferation decreased significantly by 17.7% (Figure 3A). The apoptosis assay showed that higher concentrations of Yoda1 increased the number of apoptotic MDA-MB-231 by 48.7 and 29.5% compared to DMSO at 24 h and 48 h, respectively (Figure 3B). The migration of MDA-MB-231 under Yoda1 treatments was determined using the wound closure assay. Yoda1 showed no effect on MDA-MB-231 migration under 4 h incubation, while 2, 5, and 10 μM Yoda1 significantly reduced cell migration by 16.9, 15.2, and 29.4% compared to DMSO, respectively, with a longer duration (24 h) (Figure 3C). The treatment of 1 h LMHF vibration demonstrated a trend of decreasing the migration of MDA-MB-231 but with no significant difference (Figure 3D).

After 48 h incubation, we demonstrated that the numbers of live RAW264.7 cells were significantly decreased by 62.8, 75.9, and 85.5% under 2, 5, and 10 μM Yoda1 compared to DMSO, respectively (Figure 3E). The effects of Yoda1 on osteoclast formation were also tested using a TRAP stain. We observed that 1, 2, 5, and 10 μM of Yoda1 treatments significantly reduced osteoclastogenesis of RAW264.7 after 7-day differentiation. The formation of larger TRAP-positive osteoclasts with more than 6 nuclei was reduced by 59.8, 75.9, 95.5, and 99.1% under 1, 2, 5, and 10 μM of Yoda1 compared to DMSO, respectively. The formation of osteoclasts with fewer nuclei showed no significant difference (Figure S1). Total numbers of osteoclasts were significantly reduced by 50.0, 72.5, 93.3, and 99.2% under 1, 2, 5, and 10 μM of Yoda1 treatments compared to DMSO (Figure 3F,G).

### 3.3. Combined Treatment Increased YAP Nuclear Translocation in MLO-Y4

The YAP protein plays a vital role in sensing and responding to the mechanical niche of a cell. Emerging evidence has suggested that YAP is a downstream effector of Piezo1 and mechanical loading in osteocytes [18,19,30,31]. To determine whether combining Yoda1 and LMHF vibration could increase the nuclear translocation of YAP, Western blot and immunofluorescence analyses were performed on MLO-Y4 that were stimulated by mono- (Yoda1 only or vibration only) or combined treatment (Yoda1 and LMHF vibration). The parameters for the combined treatment were chosen based on the following observations: treatment of MLO-Y4 for 1 h with 0.3 g, 60 Hz LMHF vibration was shown to significantly downregulate RANKL and inhibit osteoclastogenesis [24]; treatment of MLO-Y4 with 10 μM Yoda1 increased the intracellular calcium influx and upregulated anti-osteolytic genes [18].

**Figure 4:**
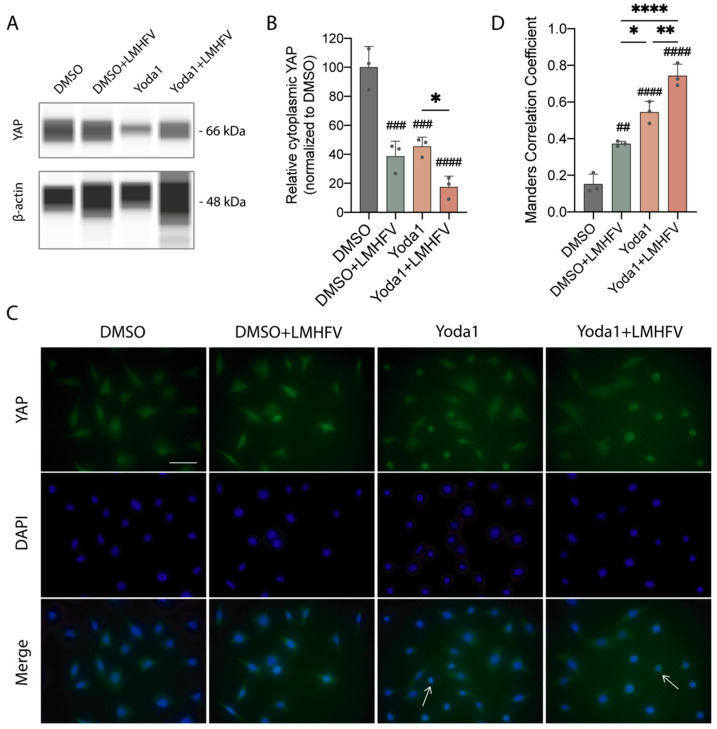
Combined treatment increased YAP nuclear translocation in MLO-Y4. (**A**) Western blot measurement of cytoplasmic YAP expression in MLO-Y4 cells stimulated with DMSO, monotreatments, or combined treatment. (**B**) Quantification of cytoplasmic YAP in cells described in (**A**). Data points represent three technical replicates (*n* = 3). (**C**) Immunofluorescent staining for YAP and nuclei (DAPI) in MLO-Y4 cells stimulated with DMSO, monotreatments, or combined treatment. Arrows indicate the representative cells with YAP localized in nucleus. Scale bar = 10 μm. (**D**) Quantification of YAP nucleus localization in cells described in (**C**) (*n* = 3). The incubation duration of Yoda1 in both Yoda1 only and combined treatment groups is 4 h. **** *p* < 0.0001, ** *p* < 0.01, * *p* < 0.05; ^####^ *p* < 0.0001, ^###^ *p* < 0.001, ^##^ *p* < 0.01 in comparison to DMSO control.

To probe the amount of cytoplasmic YAP, non-ionic detergent Triton X-100 was utilized in the lysis buffer [32,33]. The combined treatment reduced the protein expression of cytoplasmic YAP compared to individual treatments and control (Figure 4A,B and Figure S2), suggesting the synergetic effects of combined treatment in increasing nuclear translocation of YAP protein in osteocytes. The band at 66 kDa matches the molecular weight of YAP in studies that utilized the same YAP antibody [34,35,36]. Under immunofluorescence, the nucleus translocation of YAP in response to the combined treatment was significantly increased by 488.0% compared to DMSO vehicle control (Figure 4C,D). The increased nuclear translocation of YAP under combined treatment compared to the monotreatment groups was also demonstrated. 

### 3.4. Combined Treatment on Osteocytes Decreased the Formation of Osteoclasts and Further Reduced the Migration of MDA-MB-231

**Figure 5:**
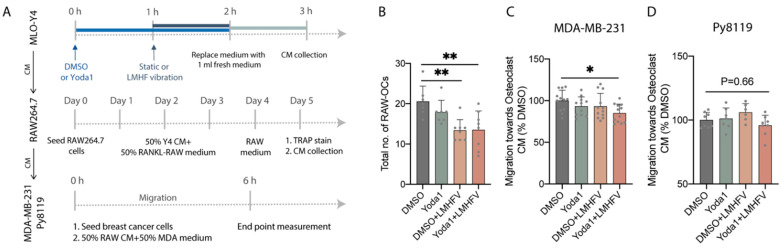
Combined treatment on osteocytes decreased osteoclastogenesis and further reduced the migration of breast cancer cells. (**A**) Experimental design of the CM study. (**B**) Total number of TRAP-positive RAW-OCs with more than 3 nuclei after 5-day differentiation under 50% CM from MLO-Y4 stimulated with DMSO, mono-treatments, or combined treatment and 50% growth medium supplemented with 20 ng/mL RANKL (*n* = 7−8). (**C**) The migration of MDA-MB-231 cells through transwell toward RAW264.7 CM (*n* = 11−12). Normalized to DMSO control. (**D**) The migration of Py8119 cells through transwell toward RAW264.7 CM (*n* = 6−8). Normalized to DMSO control. * *p* < 0.05, ** *p* < 0.01.

Since indirect signaling of flow-stimulated osteocytes to breast cancer cells through osteoclasts was shown to have anti-metastatic effects [21], we investigated whether osteocytes stimulated with LMHF vibration or the combined treatment could regulate osteoclast formation and further mediate breast cancer migration. To validate the effect of stimulated MLO-Y4 on osteoclastogenesis of RAW264.7, we first collected CM which contains secreted factors from MLO-Y4 stimulated with mono- or combined treatment (MLO-Y4 CM). Then, RAW264.7 was cultured in 50% MLO-Y4 CM and 50% growth medium that contained 20 ng/mL RANKL for 5 days. We observed a decreasing trend in forming larger osteoclasts in the combined treatment group compared to DMSO control and monotreatment groups. Furthermore, both LMHF vibration and combined treatment groups significantly decreased the formation of large osteoclasts and the total number of osteoclasts, suggesting that vibration alone on MLO-Y4 is sufficient to inhibit RAW264.7 osteoclastogenesis through CM (Figure 5B and Figure S3).

Subsequently, the migration of two breast cancer cell lines toward the CM of osteoclast cultured in stimulated MLO-Y4 CM (RAW264.7 CM) was measured using a transwell migration assay. After the invasion in response to chemoattractants for 6 h, transwell migratory of MDA-MB-231 toward RAW264.7 was slightly reduced (15.1%) compared to DMSO when MLO-Y4 cells were stimulated with combined treatment (Figure 5C). The migration of breast cancer was also slightly decreased in the monotreatment groups. However, no statistically significant difference was detected. The transwell migration of Py8119 toward RAW264.7 was also measured, but no significant difference was detected between groups (Figure 5D).

### 3.5. Breast Cancer Cells Induced Apoptosis of Osteocytes through Secreted Factors

The unidirectional CM study showed how the combined treatment applied to osteocytes affected the activity of osteoclasts and the migration of breast cancer cells. However, the effect of inverse regulation of breast cancer cells subjected to the combined treatment on osteocytes in the metastatic microenvironment remains unclear. Here, we characterized how cancer-derived soluble factors affect the total cell number, the apoptosis, and the intracellular calcium influx of osteocytes in response to oscillatory fluid flow by exposing MLO-Y4 to MDA-MB-231 CM for 3 days. 

**Figure 6:**
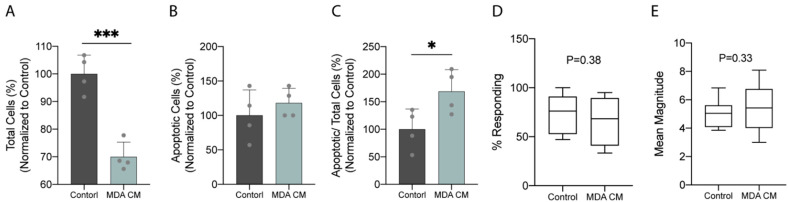
Breast cancer cells induced the apoptosis of osteocytes through secreted factors and showed no effect on the intracellular calcium influx of osteocytes. (**A**) Total MLO-Y4, (**B**) apoptotic MLO-Y4, and (**C**) apoptotic MLO-Y4 over total MLO-Y4 after 3-day incubation of MLO-Y4 in CM from non-stimulated MDA-MB-231 compared to no CM control (*n* = 4). (**D**) Intracellular calcium response percentage and (**E**) mean magnitude of MLO-Y4 under 2 Pa OFF after 3-day incubation of MLO-Y4 in CM from non-stimulated MDA-MB-231 compared to no CM control (*n* = 13 for control, *n* = 16 for MDA CM group). *** *p* < 0.001, * *p* < 0.05.

The total quantity of MLO-Y4 decreased by 30.0% after 3-day exposure to non-stimulated MDA-MB-231 CM, which is significant compared to what was observed with the non-exposure control (Figure 6A). Using the apoptosis assay, we confirmed that there is no significant difference in the number of apoptotic cells between the two groups (Figure 6B), while the percentage of apoptotic cells over the total number of cells increased significantly compared to the control (Figure 6C). In Figure 6A–C, control groups were defined as 100%, and all data were normalized to control. Despite the increased cell apoptotic rate, cancer-derived soluble factors did not affect the stimulation of calcium influx into MLO-Y4 in response to an oscillatory fluid flow of 2 Pa. The calcium response percentage (73.5% vs. 66.4%) and the mean magnitude of responding cells (5.0 vs. 5.5 times the baseline) did not differ significantly after 3-day CM exposure (Figure 6D,E).

### 3.6. Stimulation of MDA-MB-231 with Combined Treatment Decreased the Percentage of Apoptotic Osteocytes through Secreted Factors

**Figure 7:**
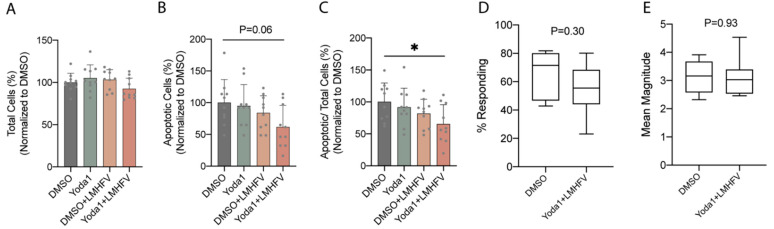
Breast cancer cells stimulated with combined treatment decreased the percentage of apoptotic osteocytes but showed no significant effect on the intracellular calcium influx of osteocytes in response to OFF. (**A**) Total MLO-Y4, (**B**) apoptotic MLO-Y4, and (**C**) apoptotic MLO-Y4 over total MLO-Y4 after 3-day incubation of MLO-Y4 in CM from MDA-MB-231 stimulated with DMSO, monotreatments, or combined treatment (*n* = 10). Normalized to DMSO control. (**D**) Intracellular calcium response percentage and (**E**) mean magnitude of MLO-Y4 under 2 Pa OFF after 3-day incubation of MLO-Y4 in CM from combined treatment stimulated MDA-MB-231 compared to DMSO (*n* = 7–8). * *p* < 0.05.

Then, we determined how the combined treatment applied to breast cancer cells modulates the total cell number and apoptosis of osteocytes. After 3-day incubation of MLO-Y4 with stimulated MDA-MB-231 CM, the total number of cells remained no different (Figure 7A), and apoptotic cells decreased by 38.2% (Figure 7B), resulting in a reduction in apoptotic MLO-Y4 over total MLO-Y4 compared to control (Figure 7C). In Figure 7A–C, DMSO groups were defined as 100%, and all data were normalized to DMSO control. Moreover, cancer cells that were subjected to combined treatment showed no significant effect on the intracellular calcium influx of MLO-Y4 in response to an oscillatory fluid flow of 2 Pa. The calcium response percentage (64.6% vs. 55.3%) and the mean magnitude of responding cells (3.1 vs. 3.1 times the baseline) did not change significantly after CM exposure (Figure 7D,E).

## 4. Discussion

**Figure 8:**
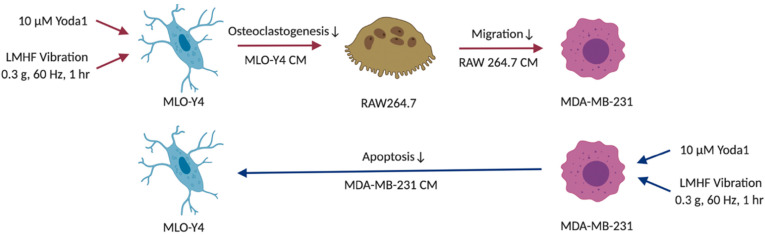
Indirect signaling from combined treatment stimulated osteocytes on breast cancer cells via osteoclasts, and the direct signaling of combined treatment stimulated breast cancer cells on osteocytes. Applying conditioned medium (CM) from combined treatment stimulated MLO-Y4 to RAW264.7 decreased the formation of larger and total number of osteoclasts. Further application of RAW264.7 CM reduced MDA-MB-231 migration. Moreover, the percentage of apoptotic MLO-Y4 decreased after subjecting to stimulated MDA-MB-231 CM. Figure was created using BioRender.

Bone metastasis is a devastating development causing major morbidity and motility for breast cancer patients. Although LMHF vibration enhances the beneficial osteogenic response, its implementation in enhancing the bone quality of breast cancer patients and postmenopausal women remains controversial. A novel combined therapy was tested in the present study, aiming to enhance osteocytes’ beneficial response to LMHF mechanical stimulation through Piezo1 activation in regulation of osteoclastogenesis and breast cancer migration. We observed supporting results for such a strategy (Figure 8). Specifically, combining LMHF vibration (0.3 g, 60 Hz, 1 h) and Yoda1 (10 μM, 2 h) showed a beneficial effect on the regulation of osteoclastogenesis and further suppressed the migration of breast cancer cells. Combined treatment increased YAP nucleus translocation in MLO-Y4, suggesting the enhanced mechanoresponse. In addition, the direct applications of Yoda1 on multiple cells were investigated. Yoda1 decreased the number of live MLO-Y4 and inhibited the proliferation and migration of MDA-MB-231. Yoda1 also showed potent regulation in inhibiting RAW264.7 viability and osteoclastogenesis. Finally, the effects of breast cancer cells under LMHFV and/or Yoda1 treatment in mediating osteocytes were investigated. MDA-MB-231 induced apoptosis of osteocytes through secreted factors, and MDA-MB-231 stimulated with the combined treatment decreased the percentage of apoptotic osteocytes through CM. 

Our study found that the application of Yoda1 has no effect in increasing the dead MLO-Y4 cells at 2, 24, and 48 h; however, it significantly decreases the number of live MLO-Y4 (Figure 2A) when treated at a high concentration (10 μM) and long duration (48 h). The interesting results might be due to the decreased proliferation of MLO-Y4 under increased Yoda1 concentration. Another interpretation is that dead MLO-Y4 cells were removed during PBS wash prior to imaging. The MLO-Y4 cultured in DMSO (0.1% *v*/*v*) for 48 h showed no effect in the number of live and dead cells compared to growth medium control (Figure S4). The significant role of Piezo1 in regulating cell survival and apoptosis has already been highlighted in numerous studies. For example, downregulation of Piezo1 reduces cell viability and proliferation in prostate cancer cells, arresting cells in the G0/G1 phase of the cell cycle [37]. Another study showed that the knockdown of Piezo1 in synovial sarcoma SW982 cells decreases cell viability, while the activation of Piezo1 using Yoda1 has no effect on cell survival [38]. In addition, a study investigating colon cancer demonstrated that Yoda1 inhibits cell viability and promotes apoptosis of HCT-116 and SW-480 cells in a dose-dependent manner [39]. This latter result aligns with our finding that Yoda1 significantly decreases the total number of MLO-Y4 (Figure 2A) and enhances the apoptosis of MDA-MB-231 (Figure 3B). 

In the present study, the migration of MDA-MB-231 cells was negatively associated with Yoda1 in a dose-dependent manner (Figure 3C). A similar effect that suggests Piezo1 negatively regulates cell migration is shown in lung epithelial cells, where the depletion of Piezo1 results in enhanced mobility and upregulated amoeboid migration marker [40]. Interestingly, Piezo1 has also been linked to promoting cell migration and motility. Blocking functional Piezo1 channels through GsMTx4 decreases the mobility of MCF-7 breast cancer cells [41]. In colon cancer cells, Yoda1 application increases cell migration, and the silencing of Piezo1 shows the opposite effect [39]. Downregulating Piezo1 through shRNA or GsMTx4 in prostate cancer results in the inhibition of migration [37]. These results suggest the importance of Piezo1 in regulating cell migration in different cell types.

Although calcium signaling plays a pivotal role in regulating osteoclast differentiation and functions [42], the regulation through Piezo1 remains largely unknown. Here, we showed that Yoda1 directly reduces cell viability of RAW264.7 at 48 h (Figure 3E). This result partially accounts for our finding that Yoda1 inhibited RAW264.7 osteoclastogenesis (Figure 3F,G). The other plausible regulation that accounts for such a finding is that Yoda1 could inhibit the differentiation of osteoclast precursors. Further studies are needed to elucidate the underlying mechanism and the role of Piezo1 in osteoclastogenesis. 

YAP, the major mechanotransducer in the cells that responds to the surrounding mechanical cues, is a critical transcriptional cofactor in controlling organ growth and suppressing tumors. YAP is normally retained in the cytoplasm and undergoes degradation; upon activation, the dephosphorylated form of YAP shuffles into the nucleus to promote transcriptional responses. Numerous studies have implicated YAP as a downstream regulator of Piezo1. Piezo1 was shown to be partially responsible for YAP nuclear translocation under fluid flow [18], and YAP was required for flow-induced expression of Ptgs2, Wnt1, and Cyr61 [18]. Moreover, YAP knockout mice showed a fragile bone phenotype, which was demonstrated similarly in Piezo1 knockout mice [30,31]. Conversely, activation of Piezo1 with Yoda1 reduced phosphorylated YAP [19], induced its nuclear translocation, and promoted targeted gene expression of the YAP pathway [18]. These studies suggest that YAP, as a Piezo1 downstream effector, plays an essential role in mechanically induced bone formation. In our study, combined treatment in MLO-Y4 significantly induced the nuclear localization of YAP from the cytoplasm (Figure 4A–D), suggesting the enhanced mechanoresponse of osteocytes. 

Although osteocytes play pivotal roles in regulating bone metastasis, they reside deeply in the mineralized bone matrix. Thus, other cells such as osteoclasts that are adjacent to the blood vessels may encounter metastatic breast cancer earlier than osteocytes during tumor progression. In this study, we confirmed that the collected CM from MLO-Y4 that were stimulated with combined treatment decreases the osteoclastogenesis of RAW264.7 (Figure 5B and Figure S3) and further inhibits the migration of MDA-MB-231 (Figure 5C) but not Py8119 breast cancer cells (Figure 5D). This suggests that the anti-osteolytic and anti-tumor potentials of the combined treatment are the results of osteocyte and osteoclast-derived factors, and the current vibration parameters have significant anti-tumor effects on MDA-MB-231 cells but a limited effect on PY8119 cells. Our results align with the previous study which showed that LMHF vibration on osteocytes regulates osteoclastogenesis through downregulation of transcriptional RANKL/OPG and decreases the amount of secreted RANKL [24]. Additionally, the concentration of intracellular calcium in MLO-Y4 was shown to significantly increase after 90 Hz of vibration for 1 h [43]. It was also previously demonstrated that mechanically stimulated osteocytes could regulate other osteoclastogenesis-inducing factors such as macrophage colony-stimulating factor (M-CSF) and C-X-C motif chemokine 10 (CXCL10) [44]. Furthermore, the migration of breast cancer cells regulated by osteoclast-derived factors has been investigated. Osteoclasts mediate tumor growth and proliferation by secreting exosomes containing microRNAs (miRNAs) such as miR-378 and miR-21 [45,46]. Other paracrine signals modulated by osteoclasts such as arachidonic acid also promote cancer metastatic features [47]. In addition to the migration of breast cancer cells, other phenotypes such as apoptosis and proliferation are clinically relevant. Since Ma et al. showed that fluid flow stimulated osteocytes and increased apoptotic breast cancer cells via osteoclasts [21], similar regulation might occur under combined-treatment-stimulated osteocytes. Further studies are needed to elucidate the secreted chemokines and other primary phenotypes under the communication of osteocytes, osteoclasts, and breast cancer cells under vibration or combined treatment.

The roles of breast cancer in modulating the apoptosis and calcium response of osteocytes were also investigated in the current study. We showed that breast-cancer-derived factors decrease the number of total cells (Figure 6A), induce apoptotic cells over total cells (Figure 6C), and have no significant effects on intracellular calcium influx of osteocytes (Figure 6D,E). The results are supported by a study on multiple myeloma which demonstrated that the interaction of myeloma cells and osteocytes increased osteocyte apoptosis [48]. Moreover, the result in intracellular calcium signal aligns with a previous finding that suggested breast-cancer-derived factors have no effect on the dendrite number of osteocytes, indicating no change in mechanosensitivity [49]. We then investigated the regulation of breast cancer cells that were stimulated with the combined treatment on osteocytes. Breast cancer cells stimulated with the combined treatment decrease apoptotic osteocytes over total cells through paracrine signals (Figure 7C). This effect can be seen primarily through the decrease in the number of apoptotic MLO-Y4, not in the total number of cells. We also demonstrate no effect on intracellular calcium influx of osteocytes in response to oscillatory fluid flow (Figure 7D,E). Though this does not align with the previous finding that mechanically loaded breast cancer cells increase the number of dendrites per cell and decrease the total number of osteocytes [49], the contribution of mechanical stimulation should be considered.

Numerous studies have investigated the mechanical regulation of cancer cells via osteocytes, mainly focusing on the direct cell-to-cell crosstalk between osteocytes and cancer cells under fluid flow stimulation. The regulation seemed flow intensity dependent [4]. Osteocytes stimulated with 1 Pa fluid flow promoted tumor migration, while stimulation under 0.25 Pa fluid flow demonstrated an altered effect. Another study showed the pro-metastatic effect of osteocytes when treated with oscillatory fluid flow (1 Pa, 1 Hz), increasing the migration and decreasing the apoptosis of breast cancer cells [21]. Additionally, it was observed in an organ-chip model that fluid flow (0.03 Pa) stimulated MLO-Y4 cells inhibited the migration of MDA-MB-231 but promoted its invasion [50]. Interestingly, when loading was applied to both breast cancer cells and osteocytes, there was an increase in the formation of dendrites in osteocytes and in the expression of E11, a gene regulating dendrite formation [49], indicating an altered mechanosensitivity. These studies show the important role of mechanical load in the direct regulation between osteocytes and breast cancer cells. The direct regulation of both cells under combined treatment will be investigated. 

In this study, we showed that the stimulation of Piezo1 using Yoda1 enhances the effects of LMHF vibration on osteocytes and breast cancer cells. However, the mechanism underlying this enhanced effect is unclear. Our observation could be interpreted through two plausible mechanisms. First, Yoda1 mimics the continuous effect of mechanical force on cells and acts as an add-on stimulation in addition to vibration. This is supported by a previous study that suggested Yoda1 in static culture could mimic the effect of laminar flow on the anti-inflammatory phenotype of endothelial cells [51]. Second, Yoda1 may reduce the threshold for mechanically stimulated channel activation. A molecular wedge mechanism for Piezo1 activation with Yoda1 was previously proposed, suggesting the binding site of Yoda1 could decouple two specific membrane domains and further increase the tension-induced extension of the arm of Piezo1 [52]. Future studies are needed to confirm the mechanism of Yoda1 in elevating the effect of mechanical stimulation.

There were several limitations in this study. First, all the supporting results were performed in vitro; further examination of combined treatment using rodent models could confirm the beneficial effects. Second, the cell lines used were derived from different species, in which MLO-Y4 osteocytes, Py8119 breast cancer cells, and RAW264.7 osteoclasts precursors are of mice origin, while MDA-MB-231 breast cancer cells are established from humans. Third, the separated upstream effects of osteocytes and osteoclasts in decreasing the migration of MDA-MB-231 are currently unable to be defined due to the experimental setups. The inhibition of osteoclastogenesis might be partially responsible for regulating the migration of MDA-MB-231. Moreover, LMHF vibration has shown to be insufficient in maintaining bone integrity for elder patients; thus, our strategy aims to elevate the beneficial effects for these patients. Here we demonstrated the enhanced mechanoresponse of osteocytes to vibration with the additional stimulation of Piezo1 using MLO-Y4 cells. Further confirmation on the effect of combined treatment using senescent osteocytes, associated with altered mechanosensitivity, is needed to recapitulate the effect in elderly patients. We expect an even more potent response of primary osteocytes isolated from elder mice to combined treatment in the regulation of bone integrity.

## 5. Conclusions

In conclusion, the combined treatment shows the effects of Yoda1 in elevating the mechanoresponse of osteocytes to LMHF vibration and further inhibiting osteoclastogenesis and MDA-MB-231 breast cancer migration. Combined treatment could be an effective option for elderly breast cancer patients at high risk of bone pain and fractures.

## Data Availability

The data presented in this study are available in this article (and Supplementary Material).

## Supplementary Materials

The following are available online at https://www.mdpi.com/article/10.3390/cancers14143395/s1, Figure S1: Number of TRAP-positive RAW-OCs with 2–3, 4–5, or more than 6 nuclei after 7-day incubation in medium (Control), DMSO, or dose-dependent Yoda1 supplemented with 20 ng/mL RANKL; Figure S2: Immunoblotting showed that combined treatment decreases the expression of cytoplasmic YAP protein in MLO-Y4; Figure S3: Number of TRAP-positive RAW-OCs with 3–5 and more than 6 nuclei after 5-day differentiation under 50% CM from MLO-Y4 stimulated with DMSO, monotreatments, or combined treatment and 50% growth medium supplemented with 20 ng/mL RANKL; Figure S4: Live-dead measurements of MLO-Y4 under growth medium (control) or 0.1% (*v*/*v*) DMSO for 48 h.
