# Supplementary material for: Yoda1 Enhanced Low-Magnitude High-Frequency Vibration on Osteocytes in Regulation of MDA-MB-231 Breast Cancer Cell Migration
Source: Cancers (Basel). 2022 Jul 13;14(14):3395. doi: 10.3390/cancers14143395 (PMC9324638; doi:10.3390/cancers14143395)
Supplement: Supplementary file 1 [file cancers-14-03395-s001.zip › cancers-1788263-supplementary.pdf]

Supplementary Materials

Yoda1 Enhanced Low-Magnitude High-Frequency Vibration on Osteocytes in Regulation of MDA-MB-231 Breast Cancer Cell Migration

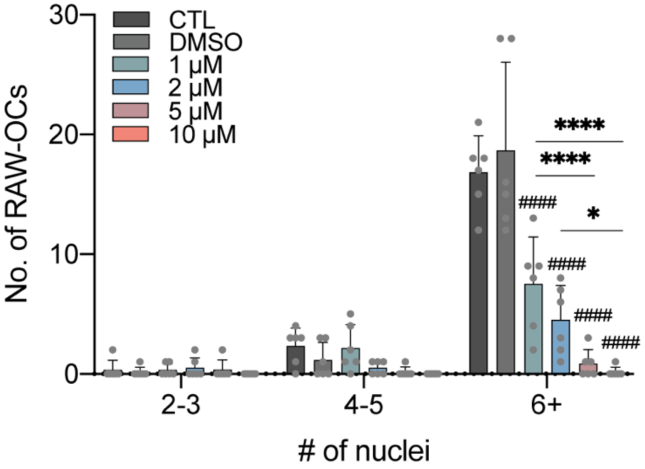

Figure S1. *Number of TRAP-positive RAW-OCs with 2-3, 4-5, or more than 6 nuclei after 7-day incubation in medium (Control), DMSO, or dose-dependent Yoda1 supplemented with 20 ng/ml RANKL (n = 6). ****P<0.0001,*P<0.5; ####P<0.0001 in comparison to DMSO control.*

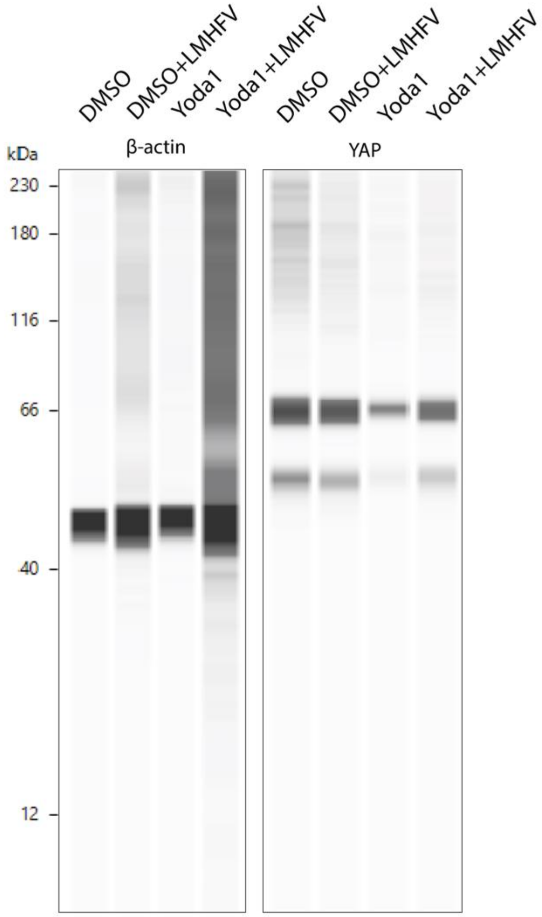

Figure S2. *Immunoblotting showed that combined treatment decreases the expression of cytoplasmic YAP protein in MLO-Y4. The uncropped images include all bands and molecular weight markers for (A) YAP protein and (B) β-actin in Fig 4A .*

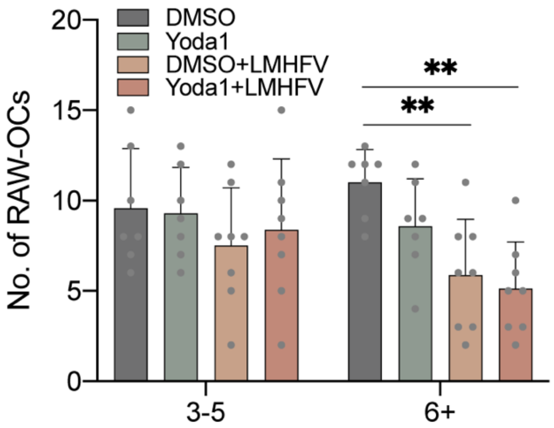

Figure S3. *Number of TRAP-positive RAW-OCs with 3-5 and more than 6 nuclei after 5-day differentiation under 50% CM frorm MLO-Y4 stimulated with DMSO, monotreatments, or combined treatment and 50% growth medium supplemented with 20 ng/ml RANKL (n=7-8). **P<0.01.*

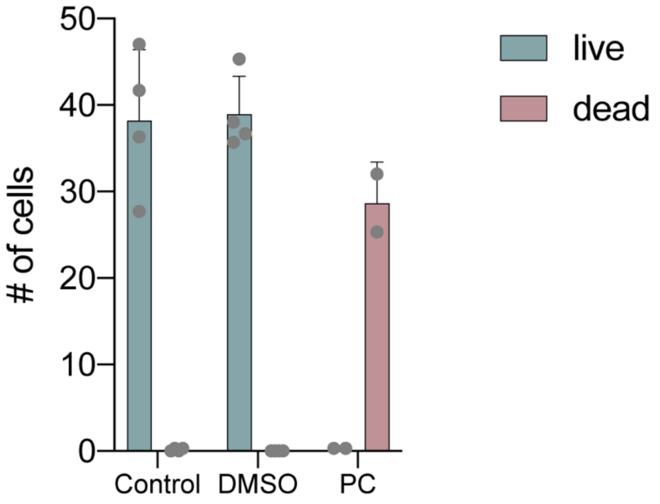

Figure S4. *Live-dead measurements of MLO-Y4 under growth medium (control) or 0.1% (v/v) DMSO for 48 h (n=4). PC: Positive control.*
